# Concentrations and Human Health Risk of Heavy Metals in Rivers in Southwest Nigeria

**DOI:** 10.5696/2156-9614-8.19.180907

**Published:** 2018-09-10

**Authors:** Ibukun Modupe Adesiyan, Mary Bisi-Johnson, Omolara Titilayo Aladesanmi, Anthony I Okoh, Aderemi Okunola Ogunfowokan

**Affiliations:** 1 Institute of Ecology and Environmental Studies, Obafemi Awolowo University, Ile-Ife, Nigeria; 2 Department of Microbiology, Obafemi Awolowo University, Ile-Ife, Nigeria; 3 SAMRC Microbial Water Quality Monitoring Centre, University of Fort Hare, Alice, South Africa; 4 Department of Industrial Chemistry, The Technical University, Ibadan, Oyo State, Nigeria; 5 Department of Chemistry, Obafemi Awolowo University, Ile Ife, Nigeria

**Keywords:** heavy metals, health, water, risk assessment

## Abstract

**Background.:**

Rivers are the most vital freshwater resources in the world. In Southwest Nigeria, anthropogenic activity stresses the quantity and quality of water resources.

**Methods.:**

The present study examined the concentrations and human health risk of five heavy metals (manganese (Mn), arsenic (As), chromium (Cr), cadmium (Cd), and lead (Pb)) in selected rivers in Southwest Nigeria. The determination of heavy metals was carried out by atomic absorption spectrophotometry after digestion with a di-acid mixture 9:4 (v/v) (nitric acid: perchloric acid).

**Results.:**

All rivers had higher concentrations of the five heavy metals in the dry season except for As in Dandaru (0.012 mg/L) and Asejire (0.016 mg/L). Manganese was observed to have the highest mean concentration among all the five metals in both the rainy and dry seasons across the sampled rivers. Generally, the annual mean concentration of metals followed the order: Mn>Cr>Cd>Pb>As in all the selected rivers.

**Discussion.:**

The human health risk assessment showed that the hazard index and hazard quotient for ingestion of water for Cd and As in all the sampled rivers were higher than the acceptable limit of 1.0, indicating carcinogenic risk (CR) via direct ingestion of water. The CR via ingestion for As in all of the sampled rivers was above the remedial goal target of 1×10^−6^. The recorded values for chronic daily intake (CDI) were higher for Cr and Mn in all four sampled rivers.

**Conclusions.:**

The results of the present study showed that As is a driver for carcinogenic risk through ingestion in all of the sampled rivers compared to other metals.

**Competing Interests.:**

The authors declare no competing financial interests

## Introduction

Rivers are the most vital freshwater resources in the world. However, increasing human development, industrialization and population growth have exerted alarming and diverse pressures on the quality, quantity and access to water resources. Toxic chemicals and heavy metals enter rivers through industrial and anthropogenic activities of urban settlement around the drainage basin of rivers. Ogunfowokan et al. stated that the types of pollutants brought into the aquatic ecosystem are largely influenced by the various anthropogenic activities taking place in the surrounding farmland.^1^ The main anthropogenic sources of heavy metal contamination are mining and smelting activities, disposal of untreated and partially treated effluents, metal chelates from different industries and indiscriminate use of heavy metal-containing fertilizers and pesticides in agricultural fields.^2^,^3^ Pollution of the aquatic environment with heavy metals has become a worldwide problem. Heavy metals are indestructible and most have toxic effects on aquatic organisms, animals and humans.^4^,^5^ Among environmental pollutants, metals are of particular concern because they are less visible, their effects on the ecosystem are intensive, they have toxic effects and bio accumulate in aquatic ecosystems, and body tissues, and organs.^6^,^7^ Heavy metals contaminate surface and ground water, resulting in deterioration of drinking water and irrigation water quality and can enter into the human food chain, posing a risk to human health.^3^,^8^ Some metals, including chromium, lead, cadmium, arsenic and mercury are known to be highly toxic to humans and aquatic life, causing liver and kidney problems in addition to genotoxic carcinogens.^9^ Others, such as copper, iron, zinc, manganese and cobalt, are essential elements which play important roles in biological metabolism at very low concentrations. The attention given to the presence of heavy metals in the environment is primarily due to their toxicity and threat to human life at elevated levels, as well as their tendency to aggravate environmental degradation and disturb the biochemical function of all environmental components, including humans and animals, necessitating regular monitoring.^1^ The aim of the present study was to evaluate the concentration of arsenic (As), lead (Pb), cadmium (Cd), chromium (Cr) and manganese (Mn) in five selected rivers in Southwest Nigeria in order to determine their human health risk.

## Methods

**Figure 1:**
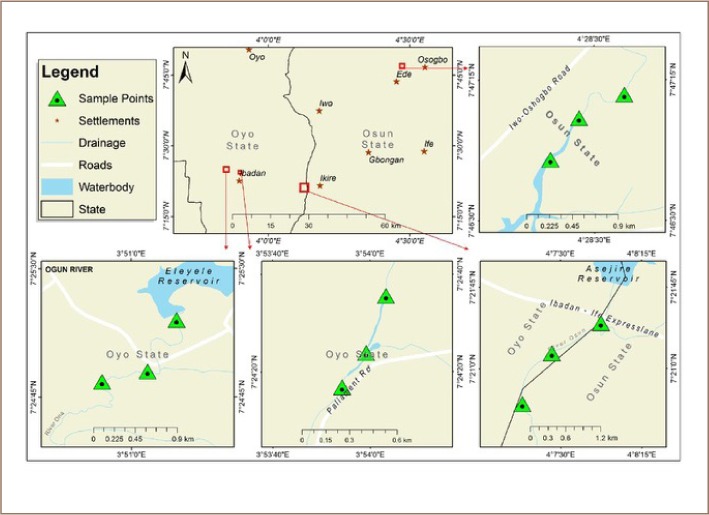
Map of study area showing sampled locations

The four river water sources sampled in this study are located in three cities in southwest Nigeria (Ibadan, Asejire and Ede) – Figure 1. These cities are traversed by rivers that function as important water supply sources, especially to the suburbs and rural areas bordering these cities. Southwest Nigeria has a tropical climate and the vegetation is predominantly semi-tropical rain forest with an average annual rainfall of about 1600 mm and average atmospheric temperature of 32°C. There are two distinct seasons: a wet season between April and October a dry season from November to March. The River Ona at Eleyele and the River Dandaru at Agodi were sampled in Ibadan, representing Oyo State. The Asejire River was sampled at the boundary of Osun and Oyo States and the Erinle River in Ede was selected to characterize Osun State. These rivers serve as collectors of industrial effluents, human and animal waste from homes, hospitals, and markets, and are often polluted with organic substances from anthropogenic activities, with higher concentrations during the dry season.

**Abbreviations**
*ADD*: Average daily dose
*CDI*: Chronic daily intake
*CR*: Carcinogenic risk
*HI*: Hazard index
*HQ*: Hazard quotient
*RfD*: Reference dose
*USEPA*: United States Environmental Protection Agency
*WHO*: World Health Organization

A number of activities take place around these rivers: markets, small scale farming, animal rearing and fishing activities, and clothes washing and bathing were also observed during sampling. The sampled rivers were coded as follows: Asejire: SR1, Dandaru: SR2, Eleyele: SR3, Erinle: SR4.

Table 1 presents the site codes, locations and a brief description of the sampled sites.

**Table 1:**
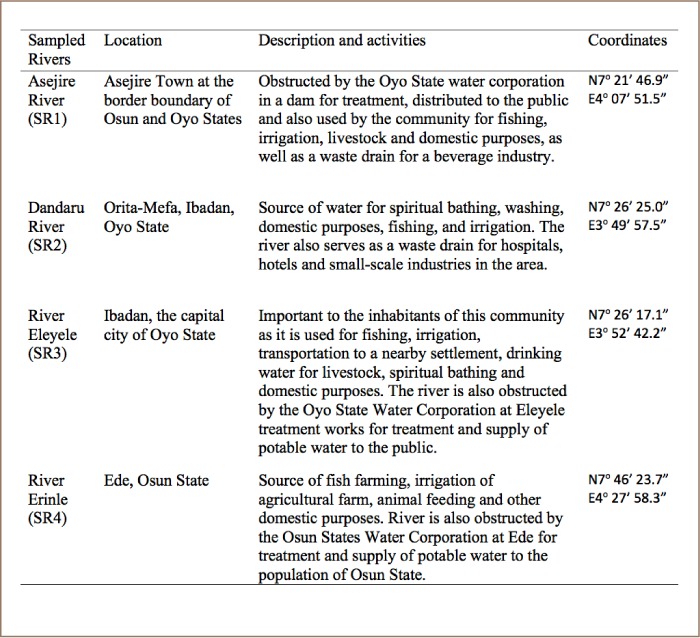
Sampling Site Description

### Sampling

Water samples were collected once a month at 3 different sampling areas in each river for a period of 12 months. Three water samples were collected from each of the four rivers monthly. A total of 144 water samples were collected at the end of the 12-month period. Water samples were collected using sterile 1 L plastic bottles. The bottles were rinsed with sample water before being filled with the sample at the sampling sites. Sampling was carried out by dipping sample bottles approximately 20 cm below the water surface, projecting the mouth of the container against the flow direction. Samples were transported in an ice chest cooler to the laboratory for analyses. Water samples were first filtered through 0.45-μm filter paper to remove particulate matter including bacteria and to slow down sample degradation.

### Heavy metal analysis

The determination of heavy metals Cd, Pb, Cr, As and Mn in sampled water was performed by atomic absorption spectrophotometry. Two hundred milliliters (200 mL) of the water sample were digested with 5 mL of 9:4 ratio v/v of di-acid mixture (nitric acid: perchloric acid) on a hot plate and filtered by Whatman No. 42 filter paper and made up to mark in a 50 mL volumetric flask by double distilled water for analysis of heavy metals using atomic absorption spectrophotometry.^10^ The concentrations obtained were compared with the European Commission, World Health Organization (WHO) and United States Environmental Protection Agency (USEPA) prescribed guidelines (*Table 2*).^11–14^

**Table 2:**
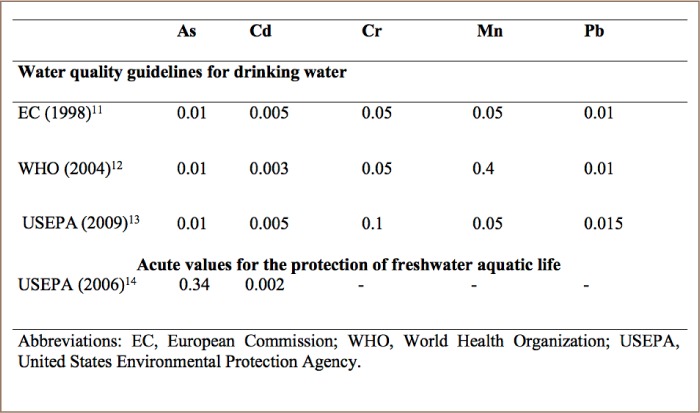
Maximum Permitted Heavy Metal Concentrations (mg/L) for Drinking Water Quality and Protection of Freshwater Aquatic Life^2^

### Human health risk assessment indices

The health risks for the heavy metals in surface water from the four sampling locations were estimated via ingestion and dermal contact based on the United States Environmental Protection Agency (USEPA) risk assessment method.^15^ The parameters for estimating human health risk assessment through different pathways are listed in Table 3. Exposure based on the average daily dose (ADD) for the heavy metals level in surface water from the four sampling locations was calculated using Equations 1 and 2, slightly modified from the USEPA protocol.^15^

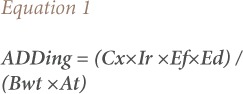
Where ADDing is the average daily dose through ingestion per kilogram of body weight.^16^,^17^ Cx is the concentration of toxic metals in drinking water (mg/L), Ir is the ingestion rate per unit time (L/day), Ed is the exposure duration (years), which is equal to the life expectancy of a resident Nigerian, Ef is the exposure frequency (days/year), Bwt is body weight (kg), and At is the averaging time (Ed x Ef). For the conversion factor from years to days, 365 days was used. The four sampled rivers assessed in this study are potential sources of drinking water for the local population, therefore water ingestion is assumed to be one of the main pathways for risk assessment. Another important pathway is dermal contact, since residents sometimes swim in these rivers and consequently may come into contact with toxic metals.^18^

**Table 3:**
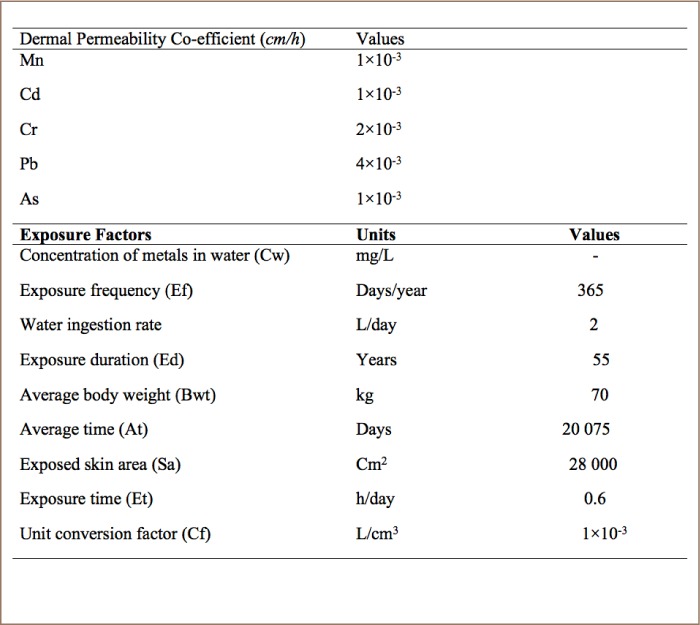
Standard Values for Calculating Exposure Assessment of Trace Metals in Surface Water Samples^15^,^17^

Average daily dose for dermal contact was therefore calculated using Equation 2.

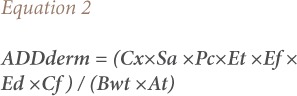
Where ADDderm is the average daily dose through dermal exposure, Sa is the total skin surface area (cm^3^), Cf is the volumetric conversion factor for water (1L/1000 cm^3^), Et is the exposure duration (h/day), Pc is the chemical-specific dermal permeability constant (cm/h), Ef is the exposure frequency (days/years), Ed is the exposure duration (years), and Bwt is body weight.

The hazard assessment was performed by comparing the calculated contaminant dose from ingestion and dermal exposure routes with the reference dose (RfD) to develop the hazard quotient (HQ) using Equation 3 below. The purpose of the hazard assessment is to evaluate whether an agent poses a non-carcinogenic hazard to humans and under what circumstances an identified hazard may be expressed.^19^

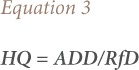
Where HQ represents the hazard quotient via ingestion or dermal contact (no units) and RfD is the oral/dermal reference dose (mg/L/day). The chronic daily intake (CDI) of the metal was estimated using Equation 4.

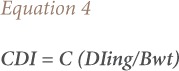
Where C is the concentration of heavy metal in water, DI is the average daily intake rate (2 L) and Bwt is body weight (70 kg).

Finally, the carcinogenic risks (CRs) of the metals were estimated using Equations 5 and 6 to assess the probability of an individual developing cancer over a lifetime as a result of exposure to a potential carcinogen. The slope factor (SF) is a toxicity value that quantitatively defines the relationship between dose and response. The SF for ingestion values ((mg/kg)/day) for As, Cd, Cr, and Pb were 1.5E+00, 6.1E+03, 5.0E+02, and 8.5E+00, respectively.^20^,^21^,^17^ Potential carcinogenic effect probabilities that an individual will develop cancer over a lifetime of exposure are estimated from projected intakes and slope factor. The range for carcinogenic risk acceptable or tolerable stipulated by the USEPA is 1×10^−6^ to 1×10^−4^.^15^

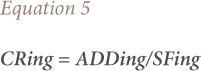


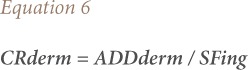
Where CRing and CRderm represent the carcinogenic risk due to ingestion and dermal exposure routes, respectively, and SF is the slope factor (mg/kg)/day. To demonstrate the lifetime carcinogenic risk that the local population may experience, the CR values was calculated for all five metals.

### Statistical analysis

Data obtained were statistically analyzed using the Statistical Package for Social Sciences (SPSS) IBM version 22 software. Both descriptive and inferential statistical analyses were carried out and used to describe the data obtained in this study. One-way analysis of variance (ANOVA) was performed to test differences among the measured parameters with respect to sampling locations. Statistical significance was set at p<0.05. In addition, cluster analysis was performed using paleontological statistics version 2.7 to identify the relationship among the analyzed metals and their possible sources.

## Results

The five heavy metals that were examined in the water samples were As, Mn, Cd, Cr and Pb. Seasonal and annual mean concentration of the five selected metals were examined in the four sampling locations as described below.

### Seasonal variation of heavy metals in Asejire River

The seasonal variation in the concentration of the five heavy metals in Asejire River (SR1) showed higher mean concentrations during the dry season for Cd (0.026 ± 0.010 mg/L), Cr (0.059 ± 0.020 mg/L), Pb (0.019 ± 0.008 mg/L) and Mn (0.211 ± 0.089 mg/L). However, As had a higher mean concentration (0.016 ± 0.020 mg/L) during the rainy season (*Table 4*). Manganese was observed to have the highest mean concentration among all the five metals in both the rainy season (0.152 ± 0.060 mg/L) and dry season (0.211 ± 0.089 mg/L) at this sampling location. There was a significant difference (p < 0.05) in the seasonal variation of Cd (P = 0.0232) and Mn (P = 0.0225), but no significant difference was observed in the seasonal variation of concentrations of Cr, Pb and As.

**Table 4:**
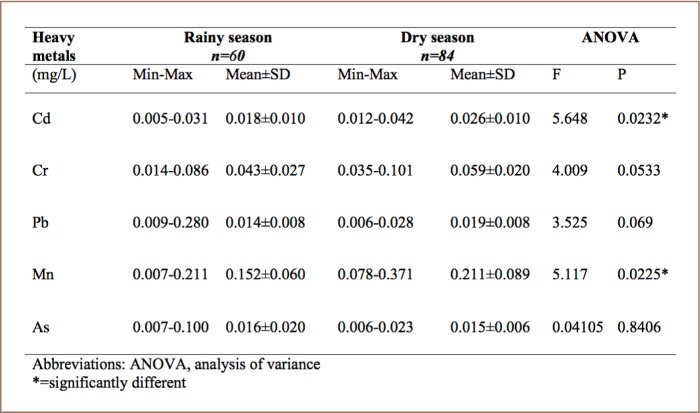
Seasonal Variation of Heavy Metals in Asejire Reservoir (SR1)

### Seasonal variation of heavy metals in Dandaru River

Samples from the Dandaru River (SR2) showed higher mean concentrations for Cd (0.024 ± 0.007 mg/L), Cr (0.071 ± 0.034 mg/L), Pb (0.019 ± 0.006 mg/L) and Mn (0.191 ± 0.073 mg/L) during the dry season than the rainy season. However, As had higher mean concentrations (0.012 ± 0.005 mg/L) during the rainy season than the dry season (*Table 5*). Manganese was also observed to have the highest mean concentrations among all metals in both the rainy season (0.162 ± 0.051 mg/L) and dry season (0.191 ± 0.073 mg/L) at this sampling location.

**Table 5:**
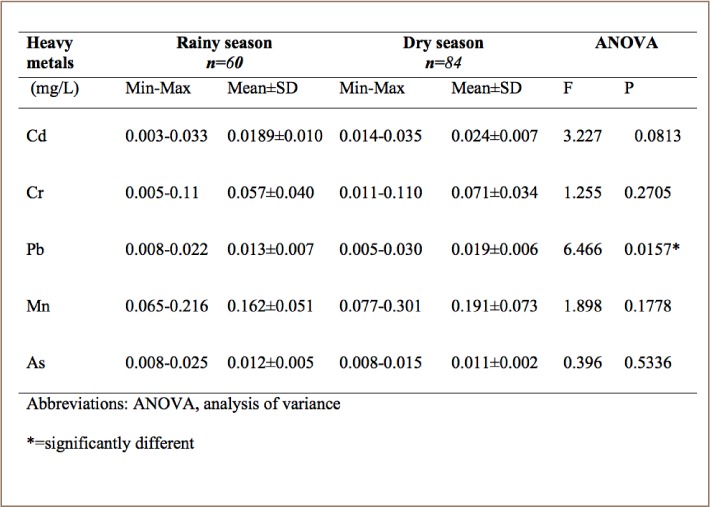
Seasonal Variation of Heavy Metals in Dandura Reservoir (SR2)

### Seasonal variation of heavy metals in Eleyele River

In the Eleyele River (SR3), mean concentrations of all of the metals were higher during the dry season: Cd (0.023 ± 0.012 mg/L), Cr (0.066 ± 0.026 mg/L), Pb (0.017 ± 0.007 mg/L), Mn (0.188 ± 0.097 mg/L) and As (0.013 ± 0.006 mg/L) (*Table 6*) than in the rainy season. Manganese had the highest mean concentrations in both the rainy season (0.159 ± 0.057 mg/L) and dry season (0.188 ± 0.097 mg/L) at this sampling location.

**Table 6:**
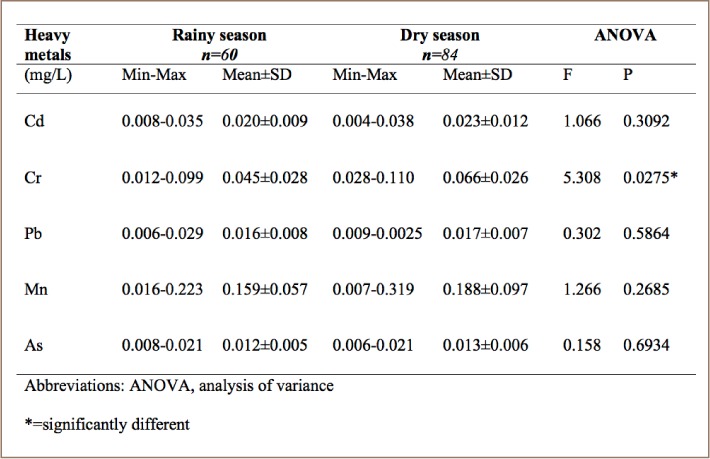
Seasonal Variation of Heavy Metals in Eleyele Reservoir (SR3)

### Seasonal variation of heavy metals in Erinle River

In Erinle River (SR4), higher mean concentrations of all the metals were recorded during the dry season as follows: Cd (0.024±0.013 mg/L), Cr (0.043±0.009 mg/L), Pb (0.022±0.007 mg/L), Mn (0.210±0.100 mg/L) and As (0.013±0.005 mg/L). Manganese had the highest mean concentrations in both the rainy season (0.175±0.056 mg/L) and dry season (0.210±0.100 mg/L) (*Table 7*).

**Table 7:**
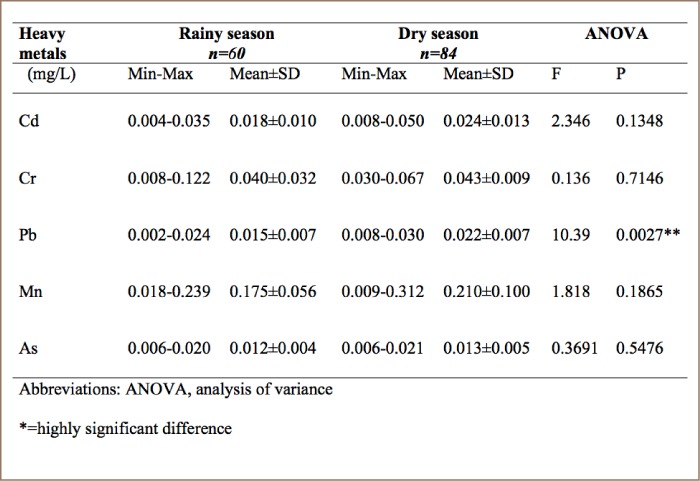
Seasonal Variation of Heavy Metals in Erinle Reservoir (SR4)

### Annual mean concentration of heavy metals across sampling locations

The annual mean concentration of Cd was highest at Eleyele River (0.022±0.009 mg/L), while Cr was highest at Dandaru with annual mean concentration of 0.063±0.031 mg/L. The highest annual mean concentrations of Pb (0.018±0.007 mg/L) and Mn (0.190±0.078 mg/L) were recorded in Erinle River. For As, the highest annual mean concentration of 0.016±0.009 mg/L was recorded at Asejire River (*Table 8*). Considering the concentration recorded for each metal at each sampling location, the following order was observed: Eleyele>Dandaru>Erinle>Asejire for Cd; Dandaru>Eleyele>Asejire>Erinle for Cr; Erinle>Eleyele>Asejire>Dandaru for Pb; Erinle>Asejire>Danda ru>Eleyele>Erinle for Mn and Asejire>Eleyele>Erinle>Dandaru for As. Generally, the annual mean concentration of metals followed the order Mn>Cr>Cd>Pb>As in all of the selected rivers. In addition, the total concentration of all of the five metals across with respect to sampling locations followed a decreasing order of Dandaru>Erinle>Asejire>Eleyele. The annual mean concentrations of metals were compared with water quality guidelines for drinking water and the protection of freshwater aquatic life and were found to exceed the guideline limits set by these regulatory bodies and this is cause for concern considering the possible deleterious effects on humans and aquatic biota.^11–14^

**Table 8:**
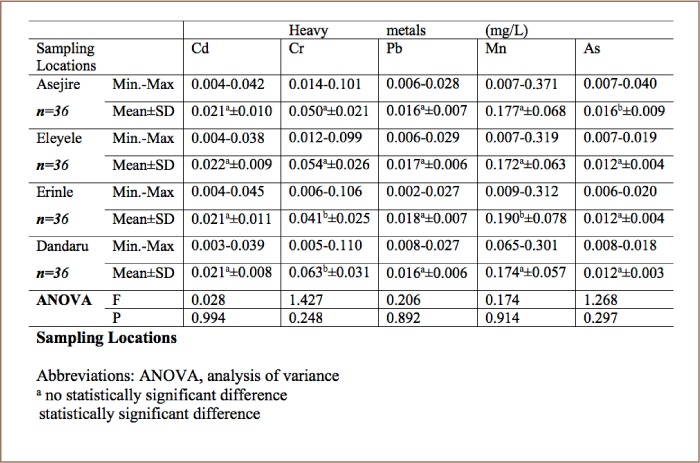
Annual Mean Concentrations (mg/L) of Heavy Metals Observed at Selected Sampling Locations

### Correlation matrix and cluster analysis

**Figure 2:**
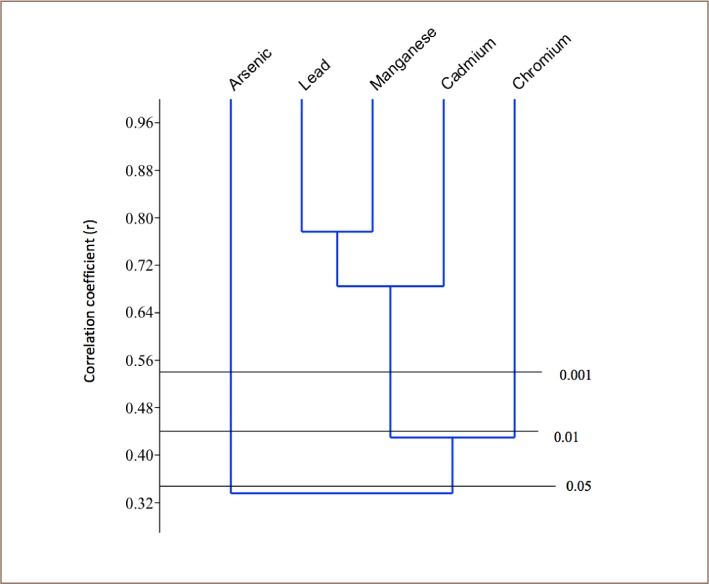
Dendogram of the Relationship among Heavy Metals in Asejire River

**Figure 3:**
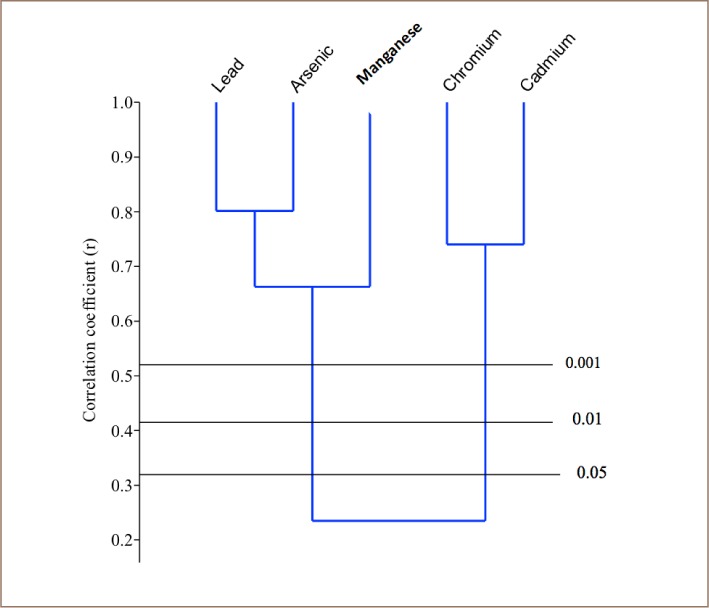
Dendogram of the Relationship among Heavy Metals in Eleyele River

**Figure 4:**
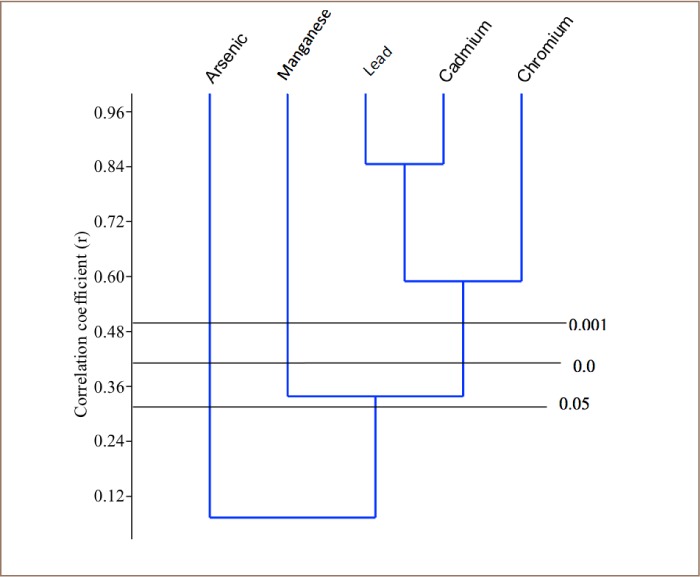
Dendogram of the Relationship among Heavy Metals in Dandaru River

**Figure 5:**
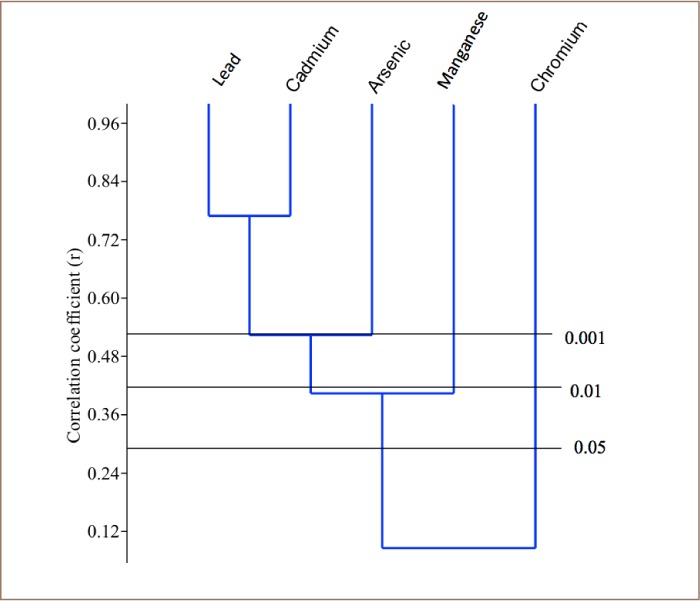
Dendogram of the Relationship among Heavy Metals in Erinle River

Cluster analysis was performed on the heavy metal results from each of the sampled rivers using paleontological statistics (PAST) version 2.7 in order to determine the relationship among the metals and their possible sources. Only one major cluster was formed in the Asejire River among the heavy metals: Pb, Mn, Cd and Cr (*Figure 2*). Two clusters were formed in the Eleyele River samples among the plotted heavy metals: Pb, As and Mn; then Cr and Cd (*Figure 3*). Only one major cluster was formed in the Dandura River (Pb, Cd, Cr) (*Figure 4*) and Erinle River (Pb and Cd) samples (*Figure 5*). The clustering pattern of these metals in each river suggests that the metals that cluster together have a common source through which they gain entry into the river. It is evidenced that all the metals originated from industrial and anthropogenic waste discharge considering their strong association with Cd.

Correlations between the analyzed parameters provided information on the relationship between the metals and their sources/pathways. The correlation matrix of heavy metal concentration from Asejire River showed that Cd had a strong positive correlation with Cr, Pb and As; in Eleyele River, Cd had strong correlation with Cr and As was strongly correlated with Mn and Pb; in Dandaru River, Cd had a strong positive correlation with Cr, Pb and Mn; while in Erinle River, Cd had strong positive correlation with Pb, Cr, and As.

### Human health risk assessment of heavy metals

The HQ for ingestion of water levels for Cd and As in all sampled rivers was higher than 1.0, signifying a high hazard effect on the local residents who utilize the river, while lower values were observed for Cr, Pb, and Mn in of all the sampled rivers. In addition, the observed values of HQ for dermal contact (*Table 9*) were lower than the 1.0 guideline in all of the sampled rivers.

**Table 9:**
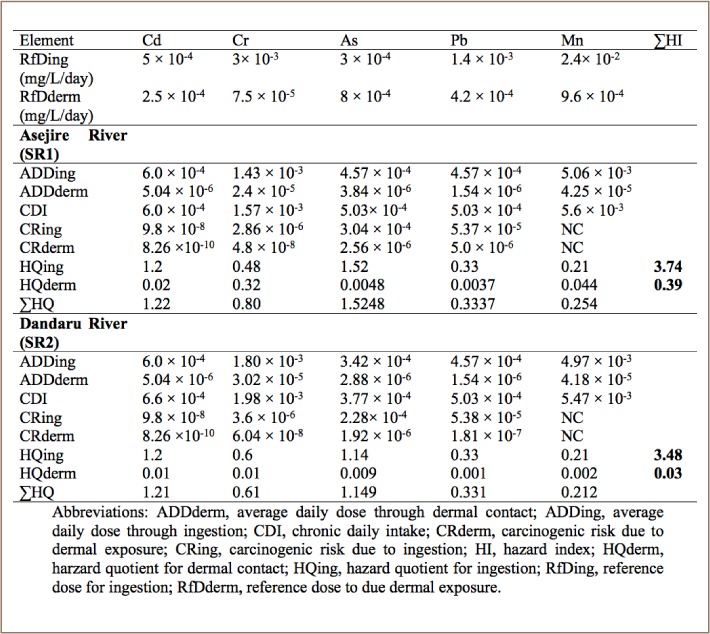
Health Risk Assessment for Metals in Water Samples from Asejire and Dandaru Rivers through Ingestion and Dermal Absorption Pathways

According to the USEPA (2004)^15^ risk assessment indices, when the value of the hazard quotient is greater than 1.0, this indicates a high probability of adverse health effects due to exposure. The ADD through ingestion and dermal contact were observed in the order Mn>Cr>Cd>Pb>As and Mn>Cr>Cd>As>Pb, respectively, in all four of the sampled rivers (*Table 9*). This indicates that Mn, Cr and Cd were major contributors to exposures through ingestion and dermal contact to those using the water from the studied rivers.

However, the computed hazard index (HI) for ingestion for all of the metals in each of the four rivers was 3.74, 3.48, 3.47, and 3.34 for Asejire, Dandaru, Eleyele, and Erinle, respectively, as shown in Tables 9 and 10, while the computed values for HI for dermal exposure were 0.39, 0.03, 0.61, 0.52 for Asejire, Dandaru, Eleyele, and Erinle Rivers, respectively. These results suggest that there is high cumulative potential of adverse health risk via direct ingestion exposure, but no cumulative potential for adverse health risk via dermal contact to the water users across all of the rivers collectively.

**Table 10:**
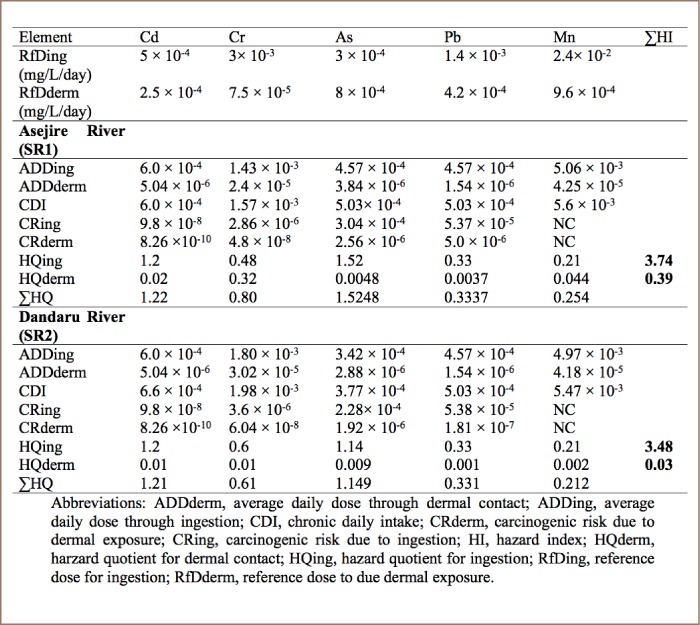
Summary of Health Risk Assessment for Metals in Water Samples from Eleyele and Erinle Rivers through Ingestion and Dermal Absorption Pathways

The CR through ingestion of metals ranged from 10^−4^ to 10^−8^, while CR due to dermal exposure ranged from 10^−6^ to 10^−10^ across all of the sampled rivers. The CR through ingestion for As in all of the sampled rivers was above the remedial goal target of 1×10^−6^. The range for carcinogenic acceptable risk is 1×10^−6^ to 1×10^−4^.^16^ The results of the present study raise carcinogenic concerns for the residents around these rivers. The results also indicate that the carcinogenic risks through ingestion of water from this river are as great as the non-carcinogenic risks.

## Discussion

Heavy metals contamination of water is a global environmental concern due to the direct impact on human health via drinking water or through consumption of contaminated aquatic organisms. The mean concentration of Cd recorded in all four studied rivers (0.021 mg/L for Asejire, 0.021 mg/L for Eleyele, 0.021 mg/L for Erinle and 0.021 mg/L for Dandura) were above the WHO maximum permissible limits for drinking water of 0.003 mg/L. The results align with similar work on three reservoirs (Ede, Opa and Asejire) which found that Cd concentrations were higher in all three reservoirs.^22^ Cadmium is a non-essential and highly toxic metal with adverse effects on living organisms.^23^ Cadmium is a chief contaminant in aquatic environments because it can easily dissolve in water.^24^ The higher concentrations of Cd recorded in all four rivers could be due to nature of the geological formation of the soil and run-off from agriculture activities where phosphate fertilizers have been applied (Cd is a common impurity in phosphate fertilizers) or as result of anthropogenic activities around the reservoir.^22^,^25^ Superphosphate and urea fertilizers are among those used by local farmers and this has been reported to contain some levels of Cd. This raises cause for concern as the use of water high in cadmium could cause adverse health effects such as renal diseases, cancer and bone pain (Itai-itai disease).^26^,^27^ Cadmium also has mutagenic and teratogenic effects.^22^

The annual mean concentrations of Pb in all of the water samples analyzed in this study (0.016 mg/L for Asejire, 0.017 mg/L for Eleyele, 0.018 mg/L for Erinle and 0.016 mg/L for Dandura) were slightly higher than the 0.01 mg/L recommended WHO maximum allowable concentration for drinking water.^28^ Lead is defined by the USEPA as potentially hazardous to most forms of life, and is considered toxic and relatively accessible to aquatic organisms even at low concentrations.^5^,^29^ Low lead concentrations affect fish by causing the formation of coagulated mucous over the gills and consequently over the entire body which results in the death of fish due to suffocation.^30^ Lead is dangerous to humans and can lead to behavioral changes and impaired performance in IQ tests.^22^ Possible sources of Pb in the rivers may be due to Pb particulate from the combustion of leaded gasoline, corrosion of lead-containing materials and burning of building and electronic wastes with residue washed into rivers.

Annual mean concentrations of Cr in the river water samples were 0.05 mg/L for Asejire, 0.054 mg/L for Eleyele, 0.041 mg/L for Erinle and 0.063 mg/L for Dandura, higher than the critical permissible level of 0.03 mg/L for drinking water recommended by the WHO and USEPA.^12^,^14^ Sources of Cr in this water sample could be due to waste consisting of lead-chromium batteries, colored polythene bags, discarded plastic materials and empty paint containers.^31^ Natural Cr compounds are generally in the trivalent state (Cr (III)); they function as micronutrients for humans and play a vital role in the metabolism of lipids and sugars.^32^ Nevertheless, anthropogenic activities can release the hexavalent form of Cr concentrations into bodies of water, which are declared carcinogenic for human health by different regulatory and non-regulatory agencies.^33^

Magnesium is introduced into aquatic environments mainly through anthropogenic sources such as sewage sludge, emissions from alloy, iron, and steel industries, municipal wastewater discharges, mining and mineral processing. Mean concentrations of manganese recorded for all the rivers were lower than the WHO provisional guideline value of 0.5 mg/L and 0.18 mg/L recommended by South African guidelines for drinking water, except in Erinle River, which is above recommended limits (0.190 mg/L).^34^ With respect to their Mn concentrations, the remaining three rivers are suitable for the survival of the aquatic ecosystem. The major effect of Mn in water for domestic use is aesthetic. Some reports have indicated that the presence of Mn in water can increase the toxicity levels of other metals, particularly Cd.^35^

The results of the present study also indicate that the As concentration was highest during the dry season in Asejire River, and had the highest annual mean concentration. The 0.01 mg/L provisional guideline value was exceeded in all of the river water samples.^12^ This may expose communities and individuals that depend on this untreated water to the risk of muscular weaknesses and cancer.^22^ These values are below the Canadian guideline value of 0.05 mg/L for the protection of fisheries and aquatic life and therefore may not adversely affect the aquatic ecosystem based on the USEPA^14^ guideline. The high level of As obtained in this study may be due to agricultural impacts on the river. Possible sources of As include residues from insecticides, herbicides and weed killers. Inorganic As compounds such as sodium arsenite have been widely used as a weed killer.^36^ Another probable source is from the burning of construction wastes such as paint cans and processed woods around the river. Arsenic is used in antifouling paints and in antifungal wood preservatives due to its germicidal power and ability to resist wood rot and decay. The discarded wastes and residues from these chemicals can interact with soil and then be washed into the rivers during rainfall. Fertilizer application to farmland around the rivers may also contribute to the increased level of observed As. Fertilizers such as superphosphate fertilizers derived mainly from phosphate ores contain significant amounts of a wide range of impurities, including trace metals such As, Cr and Cd.^37–39^ The annual mean concentrations of Cd, Cr, Pb, Mn and As were above permissible limits for drinking water, however, toxic elements like Cd, Cr and Pb occurred at levels that could pose health risks to consumer as their levels in the rivers were above the maximum allowable levels stipulated by the WHO and USEPA.^12^,^14^ Average background concentrations of Mn, Cr, Cd, Pb in soils in southwestern Nigeria are 395, 58, 0.1 and 46.4 mg/g, respectively.^40^ The background levels of heavy metals in the soil can also influence the increased concentration of these metals observed in the rivers. The activities around each of these rivers may significantly influence their metals concentrations. Farming, automobile workshops and refuse dumping are common activities around the four rivers. In Nigeria, metal scrap is a common constituent of municipal waste. According to Olanrewaju and Ilemobade, it accounts for about 1.8 % of the municipal waste generated in southwestern Nigeria.^41^ These scraps are recycled by heating, grinding and re-melting which generates dust and metal fumes in the process. Fumes generated during metal scrap recycling can also travel in the atmosphere over a long distance depending on wind speed, particle size, atmospheric temperature and humidity and then settle in soils, water and then enter humans via the food chain.^43^ Although there are metal recycling industries within the states where the study was carried out, they are located several kilometers away (40–70 km) from the studied rivers. Particles of Cd, Pb, Mn, and Cr from metal scraps that were observed to litter the area surrounding the rivers can be washed and transported during rain fall into nearby rivers leading to elevated concentrations of these metals in rivers. Fertilizer is another probable source of elevated level of metals in rivers. Studies have shown that excessive use of phosphate and urea fertilizers are one of the primary anthropogenic sources of trace metals such as As, Cr, Cd, zinc (Zn), Pb, and iron (Fe).^38^,^43^ Although the input of these metals to agricultural soil through each application of fertilizer may be small, residue deposition in water bodies through soil leaching during rainfall may result.

Generally, the annual mean concentration of metals in all of the rivers followed the toxicity order of Mn>Cr>Cd>Pb>As, similar to reports on other rivers in Southwest Nigeria, South-South Nigeria and other countries in which concentrations of all metals were above the WHO guideline limits.^44–47^ Similar studies of rivers elsewhere also reported that Mn showed the highest concentrations among studied metals.^48^,^49^ However, values of metals in the present study were higher than those of other studies.^3^,^50^,^51^ Overall, metals can be said to be the key factors impairing the river and may have serious human health implications.^52^ Strong correlations of Pb, As and Cd, as shown by their correlation matrix and clustering suggests that these metals may be coming from the same input sources. A strong correlation between As and Cd has also been reported.^17^

Human health risk assessment of heavy metals in the present study indicate that the ADD through ingestion for Cr and Mn was very high and above unity in all of the rivers. In addition, the hazard quotients for ingestion were higher than the stipulated guideline of 1.0 for Cd and As in all of the rivers, suggesting that communities exposed to this water through ingestion pathways are at risk of illness associated with consumption of high levels of these contaminants. However, the ADD and HQ from dermal exposure were low and below the threshold limit in all of the rivers, but the values for Cr and Cd were higher than other metals and require monitoring. The mean HI for dermal contact and ingestion in all of the examined rivers indicate that there was no cumulative potential for adverse health risk with regard to river water via both direct ingestion and dermal ingestion.^18^ High CDI values recorded for Cr and Mn in the four sampled rivers may indicate that agricultural and other activities around these rivers are impacting the heavy metals load in the river bodies and consequently the quality of river water.^53^,^54^ High CDI values of Cr and Mn have also been reported.^18^ The CR through ingestion of metals ranged from 2.28 × 10^−4^ to 9.8 × 10^−8^, while CR due to dermal contact ranged from 1.92 × 10^−6^ to 8.66 × 10^−10^ in all of the rivers. The CR due to ingestion and dermal contact for all of rivers were within the 1×10^−6^ to 1×10^−4^ range for carcinogenic risk acceptable by the USEPA, except for the CR due to ingestion for As which was above the remedial goal target of 1×10^−6^ in all of the sampled rivers.^15^ These results are similar to those obtained in the Yangtze river in China, the Bangshi river in Dhaka, Bangladesh and the Great Rift Valley in Kenya.^47^,^55^,^56^ The value obtained for As across all rivers raises carcinogenic concerns for the residents around these rivers.^57^ Inorganic As is a known human carcinogen. High levels of As can cause cancer of the skin, lungs, liver and bladder. Even when ingested at lower concentrations, As may cause nausea, vomiting and abnormal heart rhythm. The results also indicate that the carcinogenic risks through ingestion of river water are as high as the non-carcinogenic risks. The heavy metals analysis of the four selected rivers indicates that industrial and human activities around these rivers have adversely impacted overall water quality.

## Conclusions

The concentrations of five heavy metals (As, Cd, Cr, Pb and Mn) were determined in four rivers in Southwest Nigeria. The high concentrations of the examined heavy metals indicated that these rivers are polluted and highly impacted by surrounding industrial and anthropogenic activities. The HQ indicated potential health risk through the ingestion pathway. Furthermore, As was clearly the driver for carcinogenic risk through ingestion, as values were above the remedial goal target acceptable by the USEPA in all of the sampled rivers.^15^ It is therefore important to control increasing anthropogenic activities around these four rivers. The Nigerian Ministry of Environment should work with local authorities in each state to provide education for the resident communities around these rivers on the health impact of using untreated river water. Determination of water quality from the municipal water treatment plants using water from these rivers is also recommended.
